# Oar_Finn: the genome assembly and annotation of an exceptionally fertile Finnsheep (*Ovis aries*) Ewe

**DOI:** 10.1186/s12863-025-01354-x

**Published:** 2025-08-19

**Authors:** Juha Kantanen, Melak Weldenegodguad, Kisun Pokharel

**Affiliations:** 1 Natural Resources Institute Finland, Tietotie 4, Jokioinen, 31600 Finland; 2 Natural Resources Institute Finland, Latokartanonkaari 9, Helsinki, 00790 Finland

**Keywords:** PacBio, Fertile breed, Genetic resources, Finnsheep, Assembly, Annotation, BUSCO

## Abstract

**Objectives:**

Finnsheep, a highly prolific breed of sheep, has been globally exported for improving fertility traits of many sheep breeds. Published genomic studies of Finnsheep have been based on Texel and Rambouillet reference genomes, which may not capture its unique genetic features. Our main objective was to generate a high-quality Finnsheep genome assembly and its annotation that could serve as a breed-specific reference for studying fertility and adaptation in Finnsheep and other short-tailed northern European sheep breeds.

**Data description:**

We generated a 2.53 Gb assembly using PacBio HiFi long-reads and Hi-C sequencing from a highly fertile Finnsheep ewe. The assembly, scaffolded with Hi-C data has a contig N50 of 35.5 Mb and scaffold N50 of 100.6 Mb. Gene annotation identified 42,533 genes spanning 46.5 Mb of coding region. BUSCO completeness for the assembly and annotation was 94.9% and 84.3%, respectively. This data, including raw reads, assembly, and annotations supports genomic studies of Finnsheep and other prolific breeds of sheep that are particularly adapted to northern European environments.

## Objective

Finnsheep, the native Finnish breed of sheep (*Ovis aries*) used for meat and wool production, is among the most fertile sheep breeds globally, with an average litter size of 2.7 [5, 10, 14]. These sheep have been exported to more than 40 countries to improve fertility traits, particularly the litter size of several sheep breeds. In Finnsheep, a putative high-frequency breed-specific mutation (V371M) at *GDF9* gene has been found which may influence on high ovulation rate and litter size [14, 32]. However, fertility traits are likely polygenic, involving multiple genes and regulatory mechanisms [3, 12–14, 28]. Currently available sheep reference genomes in Ensembl (E.g., Rambouillet, Texel) represent breeds that are both morphologically and phylogenetically distinct from Finnsheep, limiting their utility for studying its unique traits [1, 2]. Finnsheep are also known for their ability to reproduce out of season, allowing them to lamb throughout the year [30]. Here we generated a Finnsheep genome assembly (Oar_Finn) from a highly fertile ewe (average litter size 5.67) using a combination of long- (PacBio HiFi) and short-read (Hi-C) sequencing approaches. The data, including raw reads, assembly, and annotations, provide a breed-specific reference for investigating fertility and adaptation in Finnsheep and related northern European short-tailed breeds. Moreover, this assembly can contribute to sheep pangenome projects, enabling broader comparative genomic studies across diverse breeds to uncover structural variations and loci related to other productivity traits. This work is part of an initiative to sequence and characterization the genomes of locally adapted native domestic breeds and semi-domestic sub-species, including Finnhorse [17], and Finnish reindeer [16].

## Data description

We sequenced the genome of a highly fertile Finnsheep ewe “Nuutilan Ihana” (flock-book number FI000025963415555, born March 3, 2012), which produced 51 lambs (46 alive) across 9 litters from 2013 to 2020, with an average litter size of 5.67. Blood was collected in EDTA tubes, and genomic DNA was extracted using the Qiagen Blood and Cell Culture Midi Kit (Qiagen, Hilden, Germany). DNA was quantified using Qubit 2.0 Fluorometer (Life Technologies, Carlsbad, CA, USA). Four PacBio SMRTbell libraries (~ 20 kb) were prepared using the SMRTbell Express Template Prep Kit 2.0 (PacBio, Menlo Park, CA, USA) and sequenced on the PacBio Sequel. A total of 10,066,601 HiFi reads comprised 299.5 Gb representing > 100x coverage with an average sequence length of 29,752 bp and N50 length of 45,920 bp. A Dovetail Hi-C library was prepared following the established protocols [9] and sequenced on a single lane of an Illumina HiSeq X yielding 908,819,867 paired-end (2 × 150 bp) reads.

The initial assembly was generated using Wtdbg2 [29] with default parameters. A fraction of the scaffolds was identified as contaminant and were removed from the assembly. Haplotypic duplications were purged with purge_dups v1.1.2 [6] producing a 2.53 Gb primary assembly with a conting N50 of 35.5 Mb. Scaffolding with HiRise [18] using Hi-C reads improved contiguity, resulting in a N50 contig and N50 Scaffold lengths of 47.7 Mb, and 100.6 Mb, respectively (Table 1, Data files 1 and 2). PacBio subreads were used for K-mer analysis using KMC3.2.4 [8]. GenomeScope profile [27] revealed the genome size to be 2.58 Gb, therefore our assembly represents 98% of the estimated genome size (Table 1, Data file 3). BUSCO comparison [11] against the eukaryota dataset showed that the assembly had 95% completeness (Table 1, Data file 2).

Gene annotation employed an ab initio approach with MAKER v2.31.10 [7], SNAP v2006-07-08 [33], and AUGUSTUS v2.5.5 [31]. Coding sequences from *Ovis aries*,* Capra hircus*,* Bos taurus*, and *Sus scrofa* were used to train the initial ab initio models. RepeatModeler v2.0.1 revealed that 41.28% of the genome was comprised of repeats, primarily class I TE (33.1%) repeats. A total of 42,533 genes were predicted and coding regions of the assembly comprised 46.5 Mb (Table 1, Data files 4 and 5). BUSCO analysis on the predicted genes indicated 84.3% completeness. We annotated 55 genes including fertility genes (E.g., *BMP15*, *GDF9*, *BMPR1B*) and *FecL* [4, 15] (Table 1, Data file 6). Interestingly, the V371M mutation in *GDF9*, previously reported in Finnsheep and linked to high fertility [14], was absent in the assembly. This absence suggests new insights into the genetics of fertility in Finnsheep, indicating that other genetic factors may be contributing to this trait. Chromatin analysis identified 55 topologically associating domains (TADs) with 335 in A compartments and 330 in B compartments (Table 1, Data file 7).

**Table 1:** Overview of data files/data sets

Label	Name of data file/data set	File types (file extension)	Data repository and identifier (DOI or accession number)
Data file 1	Oar_Finn: Genome assembly of Finnsheep ewe	Fasta file (fasta.gz)	European Nucleotide Archive (http://identifiers.org/assembly:GCA_965637775) [19]
Data file 2	Finnsheep reference genome (Oar_Finn): assembly statistics	MS Word file (.docx)	Figshare (10.6084/m9.figshare.29152808.v2) [20]
Data file 3	Finnsheep reference genome (Oar_Finn): GenomeScope results	MS Word file (.docx)	Figshare (10.6084/m9.figshare.29153150.v1) [21]
Data file 4	Finnsheep reference genome (Oar_Finn): genome annotation statistics	MS Word file (.docx)	Figshare (10.6084/m9.figshare.29152934.v1) [22]
Data file 5	Finnsheep reference genome (Oar_Finn): genome annotation file	General feature format (.gff)	Figshare (10.6084/m9.Figshare.29581943) [23]
Data file 6	Finnsheep reference genome (Oar_Finn): list of manually annotated genes	Portable document format (.pdf)	Figshare (10.6084/m9.figshare.29469176.v1) [24]
Data file 7	Finnsheep reference genome (Oar_Finn): TAD report	MS Word file (.docx)	Figshare (10.6084/m9.figshare.29153168.v1) [25]
Data file 8	Finnsheep reference genome (Oar_Finn): comparison of key metics with other fertile breeds	MS Word file (.docx)	Figshare (10.6084/m9.figshare.29469212.v1) [26]

## Limitations

While the statistics of our genome assembly is on par with existing reference genomes (Table 1, Data file 8), and the data can still provide valuable insights into the genetic makeup of Finnsheep. Incorporating ultra-long reads (e.g., Oxford Nanopore) could resolve repetitive regions and improve contiguity. PacBio subreads used for k-mer analysis have a relatively high error rate (9.8% as indicated by GenomeScope), which introduces noise in k-mer counts compared to HiFi reads and might have affected the accuracy of genome size estimation and heterozygosity assessment.

## Data Availability

The genome assembly and annotation data described in this Data note can be freely and openly accessed at http://identifiers.org/assembly: GCA_965637775. Please see Table 1 for details and links to the data.
